# RNA-Based Strategies for Cardiac Reprogramming of Human Mesenchymal Stromal Cells

**DOI:** 10.3390/cells9020504

**Published:** 2020-02-22

**Authors:** Paula Mueller, Markus Wolfien, Katharina Ekat, Cajetan Immanuel Lang, Dirk Koczan, Olaf Wolkenhauer, Olga Hahn, Kirsten Peters, Hermann Lang, Robert David, Heiko Lemcke

**Affiliations:** 1 Department of Cardiac Surgery, Reference and Translation Center for Cardiac Stem Cell Therapy (RTC), Rostock University Medical Center, 18057 Rostock, Germany; Paula.Mueller@uni-rostock.de (P.M.); Heiko.Lemcke@med.uni-rostock.de (H.L.); 2 Faculty of Interdisciplinary Research, Department Life, Light & Matter, University Rostock, 18059 Rostock, Germany; 3 Institute of Computer Science, Department of Systems Biology and Bioinformatics, University of Rostock, 18057 Rostock, Germany; Markus.Wolfien@uni-rostock.de (M.W.); Olaf.wolkenhauer@uni-rostock.de (O.W.); 4 Department of Cell Biology, Rostock University Medical Center, 18057 Rostock, Germany; Katharina.Ekat@med.uni-rostock.de (K.E.); olga.hahn@med.uni-rostock.de (O.H.); Kirsten.Peters@med.uni-rostock.de (K.P.); 5 Department of Cardiology, Rostock University Medical Center, 18057 Rostock, Germany; Cajetan.lang@med.uni-rostock.de; 6 Institute of Immunology, Rostock University Medical Center, 18057 Rostock, Germany; Dirk.Koczan@med.uni-rostock.de; 7 Stellenbosch Institute of Advanced Study, Wallenberg Research Centre, Stellenbosch University, 7602 Stellenbosch, South Africa; 8 Department of Operative Dentistry and Periodontology, Rostock University Medical Center, 18057 Rostock, Germany; Herman.Lang@med.uni-rostock.de

**Keywords:** mesenchymal stromal cells (MSC), mRNA, miRNA, cardiac reprogramming, cardiac differentiation

## Abstract

Multipotent adult mesenchymal stromal cells (MSCs) could represent an elegant source for the generation of patient-specific cardiomyocytes needed for regenerative medicine, cardiovascular research, and pharmacological studies. However, the differentiation of adult MSC into a cardiac lineage is challenging compared to embryonic stem cells or induced pluripotent stem cells. Here we used non-integrative methods, including microRNA and mRNA, for cardiac reprogramming of adult MSC derived from bone marrow, dental follicle, and adipose tissue. We found that MSC derived from adipose tissue can partly be reprogrammed into the cardiac lineage by transient overexpression of GATA4, TBX5, MEF2C, and MESP1, while cells isolated from bone marrow, and dental follicle exhibit only weak reprogramming efficiency. qRT-PCR and transcriptomic analysis revealed activation of a cardiac-specific gene program and up-regulation of genes known to promote cardiac development. Although we did not observe the formation of fully mature cardiomyocytes, our data suggests that adult MSC have the capability to acquire a cardiac-like phenotype when treated with mRNA coding for transcription factors that regulate heart development. Yet, further optimization of the reprogramming process is mandatory to increase the reprogramming efficiency.

## 1. Introduction

Mesenchymal stromal cells (MSC) represent a multipotent cell population capable to differentiate into different cell types [1]. They are an easily-accessible cell source as they can be isolated at high yields from various kinds of human tissue, such as umbilical cord, bone marrow, dental pulp, adipose tissue, placenta, etc. [1]. The common mesenchymal cell types that emanate from MSC are osteocytes, chondrocytes, and adipocytes [2]. Due to their plasticity, MSC are considered as one of the most important cell types for the application in regenerative medicine as demonstrated by a huge number of pre-clinical studies and several clinical trials [3,4]. In addition, MSC mediate immunomodulatory and immunosuppressive effects that promote wound healing and tissue repair, while showing no teratoma formation post transplantation [5]. Nowadays, it is commonly accepted that the observed therapeutic impact induced by MSCs is mainly based on the secretion of paracrine factors rather than on the differentiation into cardiomyocytes.

In recent years, MSC have also been utilized for the generation of mesenchymal as well as non-mesenchymal cell lineages, including neuron-like, hepatocyte-like, and cardiac-like cells [6,7,8,9,10]. Despite these promising results, the differentiation of human MSC into fully mature cardiomyocytes bearing all their respective phenotypical and functional characteristics is difficult [11,12,13,14,15]. As MSC are located in various tissues, they represent a heterogeneous progenitor cell population dependent on the tissue source and the individual donor [16]. This heterogeneity could explain the variety in differentiation characteristics [17,18,19]. Therefore, it remains to be investigated which type of MSC favorably undergoes cardiac trans-differentiation, thus, is a suitable candidate for cardiac reprogramming strategies. Detailed knowledge about the cardiac differentiation potential of specific MSC populations is even more important as some studies showed enhanced therapeutic effects following cardiovascular lineage commitment of MSC [12].

The development of an approach to efficiently control the cardiac differentiation of MSC would be a crucial step for the production of patient-derived cardiomyocytes without any ethical concerns. As such, they can also serve as a model system, beneficial for basic cardiovascular research, drug screening, and translational applications. Currently, several re/programming strategies exist to guide the mesenchymal and non-mesenchymal differentiation of MSC, such as treatment with small molecules and cytokines, exposure to metabolic stress, co-culture experiments, or overexpression of regulatory proteins [20,21,22,23,24]. For the potential clinical use, transient, non-integrative reprogramming approaches are preferred to prevent permanent alterations of the genome and to reduce tumorigenic risk. Small non-coding RNAs, like microRNAs (miRNA) and chemically modified messenger RNA (mRNAs) allow the manipulation of cell behavior for a limited period of time, e.g., triggering (trans)-differentiation by activation of lineage-specific molecular pathways. Some studies have already shown that alteration of gene expression using selected miRNAs can induce cardiac differentiation of MSC to a small extent [15,25,26], while data about mRNA-based cardiac reprogramming is still lacking.

Unlike multipotent MSCs, pluripotent stem cells (PSCs) have been demonstrated to efficiently differentiate into cardiomyocytes, characterized by a profound sarcomere organization and spontaneous beating behavior [27]. Yet, these PSC-derived cardiomyocytes typically still represent an immature cell type, resembling a neonatal cell stage rather than an adult phenotype [28,29]. The common cardiac programming approaches used to guide cardiac differentiation of PSCs mainly relies on the application of cytokines and small molecules [30,31]. However, PSCs bear tumorigenic risk due to genome modification (induced pluripotent stem cells, iPSC) and provoke ethical concerns (embryonic stem cells, ESC). Therefore, increasing the efficiency of cardiac programming of MSC would be beneficial for cardiovascular research, including their therapeutic use.

Here, we examined whether MSC derived from different sources, including bone marrow (BM), dental follicle and subcutaneous adipose tissue can be driven towards a cardiac lineage using a transient reprogramming strategy based on miRNA and mRNA transfection. According to our results, adipose tissue-derived MSC (adMSC) were found to be the most susceptible cell type for this reprogramming approach, as shown by enhanced expression of cardiac markers. At the same time, we observed the activation of transcriptome pathways involved in cardiac development following mRNA treatment.

## 2. Material and Methods

### 2.1. Cell Culture

BM-derived MSC (BM MSC) were obtained by sternal aspiration from donors undergone coronary bypass graft surgery. Anticoagulation was achieved by heparinization with 250 i.E./mL sodium heparin (Ratiopharm, Ulm, Germany). Mononuclear cells were isolated by density gradient centrifugation on 1077 Lymphocyte Separation Medium (LSM; PAA Laboratories, Pasching, Germany). MSC were enriched by plastic adherence and sub-cultured in MSC basal medium supplemented with SingleQuot (all Lonza, Cologne, Germany) and 1% Zellshield (Biochrom, Berlin, Germany).

Isolation of adMSC was performed by liposuction of healthy individuals. The extracted tissue was treated with collagenase for 30 min, followed by several filtrations and washing steps. The detailed process of adMSC isolation has been already described previously [32].

Dental follicle stem cells (DFSCs) were isolated from dental follicles of extracted wisdom teeth before tooth eruption. Following tooth removal, the follicle was removed and subjected to enzymatic treatment as presented earlier [33]. Upon tissue digestion, cells were seeded on tissue flasks and obtained by plastic adherence. DFSCs were maintained in DMEM-F12 (Thermo Fisher, Waltham, USA) supplemented with 10% FCS and 1% Zellshield.

All three types of stromal cells were maintained at 37 °C and 5% CO_2_ humidified atmosphere. Medium was changed every 2–3 days. Sub-cultivation was performed when cells reached a confluency of ~80–90%.

All donors have given their written consent for the donation of their tissue according to the Declaration of Helsinki. The study was approved by the ethical committee of the Medical Faculty of the University of Rostock (registration number: bone marrow A2010-23; renewal in 2015; adipose tissue: A2013-0112, renewal in 2019, dental tissue: A 2017-0158).

### 2.2. Fluorescence-Activated Cell Sorting

The expression of cell surface markers was quantified by flow cytometric analysis. Stromal cells were labelled with antibodies CD29-APC, CD44-PerCP-Cy5.5, CD45-V500, CD73-PE, CD117-PE-Cy7, PerCP-Cy5.5 CD90 (BD Biosciences, San Jose, USA), and CD105-AlexaFluor488 (AbD Serotec, Oxford, UK). Respective isotype antibodies served as negative controls. A measurement of 3 × 10^4^ events was carried out using BD FACS LSRII flow cytometer (BD Biosciences).

To evaluate miRNA and mRNA uptake efficiency, cells were treated with different amounts of Cy3-labeled Pre-miRNA Negative Control #1 (AM17120, Thermo Fisher) or GFP-mRNA (Trilink, San Diego, USA) and analyzed by flow cytometry 24 h post transfection. To detect cytotoxicity, cells were labelled with Near-IR LIVE/DEAD fixable dead cell stain kit (Molecular Probes, Eugene, USA). Analysis of flow cytometry data, including gating, was conducted with the FACSDiva software, Version 8. (Becton Dickinson).

### 2.3. Cardiac Reprogramming

For cardiac reprogramming, 1 × 10^5^ cells/well were seeded on 0.1% gelatin-coated 6 well plates and cultured to 80% confluency. We transfected 40 pmol of each miRNA (Pre-miR™ hsa-miR-1, Pre-miR™hsa-miR-499a-5p, Pre-miR™hsa-miR-208a-3p, Pre-miR™hsa-miR-133a-3p, all Thermo Fisher) with Lipofectamine^®^ 2000 according to the manufacturer’s instructions (Thermo Fisher). Transfection of custom-made mRNA (Trilink) was performed with Viromer Red^®^ transfection reagent (Lipocalyx, Halle, Germany). Cells were either transfected with 2 µg MESP1 or with a combination of 1 µg GATA4, 1 µg MEF2C and 1 µg TBX5. One day after transfection of miRNA or mRNA, cells were subjected to two different medium conditions. For cardiac induction medium I (card ind. I), cells were incubated in RPMI, supplemented with B27 without insulin (Thermo fisher) for 7 days, followed by incubation in RPMI containing B27 +insulin/- vitamin A (Thermo Fisher) for another 21 days. Additionally, culture medium was supplemented with ascorbic acid (Sigma Aldrich, St. Louis, USA) and Wnt pathway targeting small molecules, including 6 µM CHIR99021 (days 1–2), and 5 µM IWP-2 (days 4–5) (both Stemcell Technologies). For cardiac induction II (card ind II), a commercially available cardiomyocyte differentiation kit was used according to the instructions given by the manufacturer (Thermo Fisher, A2921201).

### 2.4. IF Staining and Calcium Imaging

To verify multipotency, BM-MSC, DFSC and adMSC were subjected to in vitro differentiation towards osteogenic, chondrogenic and adipogenic lineages using the Mesenchymal Stem Cell Functional Identification Kit (R & D). Differentiation was induced by maintaining cells under different culture conditions according to the manufacturer instructions for 20 days. Subsequently, cells were fluorescently labelled to detect fatty acid-binding protein 4 (FABP4), Aggrecan and Osteocalcin to visualize successful differentiation into adipocytes, chondrocytes, and osteocytes.

For labelling of cardiac markers, cells were seeded on coverslips and fixed with 4% PFA. Antibody staining was performed as described elsewhere [34]. Cells were labelled with anti sarcomeric α-actinin (abcam, ab9465), anti-NKX2.5 (Santa Cruz, sc-8697), anti-TBX5 (abcam, ab137833) and anti-MEF2C (Santa Cruz, sc-313).

To visualize intracellular calcium, cells were cultured on 8 well chamberslides (Ibidi). Three days after seeding, cells were incubated with the calcium sensitive dye Cal520 (AATBioquest) for one hour at 37 °C and subjected to fluorescence microscopy. All fluorescence images were acquired using Zeiss ELYRA LSM 780 (Zeiss, Oberkochen, Germany).

### 2.5. RNA Isolation and Quantitative Real-Time Polymerase Chain Reaction

Isolation of cellular RNA was performed using the NucleoSpin^®^ RNA isolation kit (Macherey-Nagel, Düren, Germany) according to the manufacturer instructions. The concentration and purity of isolated RNA was assessed with NanoDrop 1000 Spectrophotometer (Thermo Fisher Scientific). Subsequently, cDNA synthesis was performed with a High-Capacity cDNA Reverse Transcription Kit (Thermo Fisher Scientific). The reverse transcription reaction was conducted using the MJ Mini™ thermal cycler (Bio-Rad).

Quantitative real-time PCR for cardiac marker genes was carried out using the StepOnePlus™ Real-Time PCR System (Applied Biosystems, Foster City, USA) with following reaction parameters (StepOne™ Software Version 2, Applied Biosystems, Germany): start at 50 °C for 2 min, initial denaturation at 95 °C for 10 min, denaturation at 95 °C for 15 s and annealing/elongation at 60 °C for 1 min with 40 cycles. A qPCR reaction contained: TaqMan^®^ Universal PCR Master Mix (Thermo Fisher), respective TaqMan^®^ Gene Expression Assay, UltraPure™ DNase/RNase-Free Distilled Water (Thermo Fisher), and 30 ng of the respective cDNA. The following target gene assays were used: ACTN2 (Hs00153809_m1); MYH6 (Hs01101425_m1) TBX5 (Hs00361155_m1); TNNI3 (Hs00165957_m1), GJA1 (Hs00748445_s1); HPRT (HS01003267_m1) (all Thermo Fisher). Obtained CT values were normalized to HPRT and data were calculated as fold-change expression, related to untreated control cells.

### 2.6. Microarray Analysis

RNA integrity was analyzed using the Agilent Bioanalyzer 2100 with the RNA Pico chip kit (Agilent Technologies). 200 ng of isolated RNAs were subjected to microarray hybridization as described in [35]. Hybridization was performed on Affymetrix Clariom^TM^ D Arrays according to the manufacturer’s instructions (Thermo Fisher).

Analysis of the microarray data was conducted with the provided Transcriptome Analysis Console Software from Thermo Fisher (Version 4.0.1, Waltham, USA). The analysis included quality control, data normalization, and statistical testing for differential expression (Limma). Transcripts were considered as significantly differentially expressed with a fold change (FC) higher than 2 or smaller −2, false discovery rate (FDR) < 0.05, and *p* < 0.05. The pathway analyses were conducted based on a gene set enrichment analysis using Fisher’s Exact Test (GSEA) on the Wiki-Pathways database. Only significant pathways have been selected.

### 2.7. Statistical Analysis

Data are presented as mean ± SEM, obtained from three patients for each MSC type. Preparation of graphs and statistical analysis was performed using SIGMA Plot software (Systat Software GmbH, Erkrath, Germany). Statistical significance was considered as * *p* ≤ 0.5, ** *p* ≤ 0.05, *** *p* ≤ 0.001.

## 3. Results

### 3.1. Characterization of Isolated MSC

**Figure 1:**
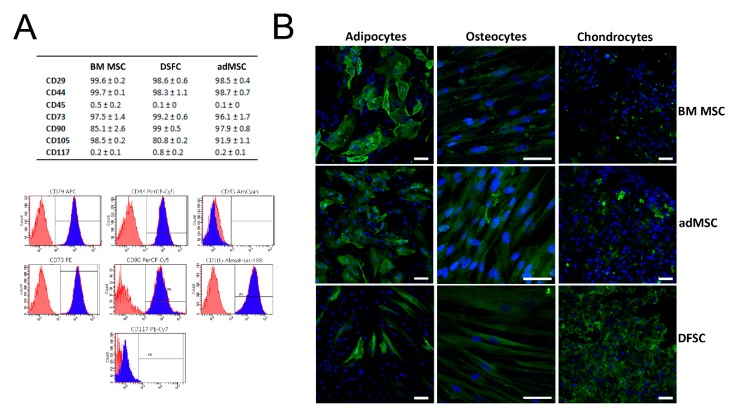
Phenotype-related and functional characterization of mesenchymal stromal cells (MSC): (**A**) Flow cytometric measurements revealed a high expression of common MSC surface markers (CD29, CD44, CD73, CD90, CD105), while very low levels were found for hematopoietic surface markers (CD45 and CD117). Representative flow cytometry charts of adipose tissue-derived MSC (adMSC) demonstrate the expression level of surface markers. Blue histograms represent measurement of CD surface marker with corresponding isotype control, shown in red. (**B**) Tri-lineage differentiation assay indicated adipogenic, osteogenic, and chondrogenic differentiation of MSC. Detection of adipocytes was performed by labelling of FABP4, while osteocytes and chondrocytes were identified by fluorescence staining of osteocalcein and aggrecan, respectively. Scale bar: 50 µm. Results in (**A**) are shown as mean ± SEM, obtained by analysis of three different donors for each MSC cell type.

Initially, we performed flow cytometric analysis to investigate the presence of common mesenchymal surface markers in isolated MSC. The obtained data indicated a high expression of CD29, CD44, CD73, CD105 and CD90, while very low levels were detected for CD117 and CD45, indicating that stem cells possess properties of MSC (Figure 1A,B).

MSC characteristics were further confirmed by a functional assay that demonstrated the multilineage differentiation capability of all three cell types. Upon incubation in lineage-specific induction medium, the cells were capable to differentiate into adipocytes, chondrocytes, and osteocytes, as shown by fluorescence labelling of specific differentiation markers (Figure 1B). As expected, adMSC were found to profoundly express FABP4, if compared to osteocalcin and aggrecan labelling. In contrast, DFSCs favored chondrogenic differentiation indicated by strong fluorescence intensity of aggrecan staining.

**Figure 2:**
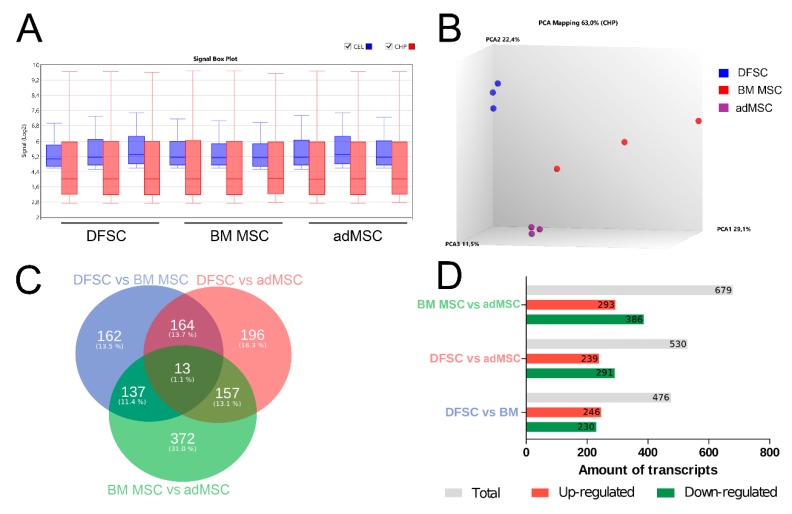
Comparative microarray analysis of undifferentiated dental follicle stem cells (DFSCs), bone marrow (BM) MSC, and adMSC. (**A**) Comparison of signal intensity for .cel files (blue) and .chp files (red) after normalization demonstrates sufficient data quality. (**B**) MSC from different sources are clearly distinct in regard to their transcription profile. A high patient-dependent variety was found for BM MSC, while adMSC and DFSCs demonstrate a more homogenous distribution. (**C**) Venn diagram visualizes expressed genes overlapping between different MSC cell types. (**D**) The numbers of up- and down-regulated transcripts is significantly differentially expressed in all three cell types.

Next, we compared the different MSC by analyzing their gene expression profiles using a microarray platform. The obtained data allowed us to compare the transcription profile among both, individual donors and MSC derived from different tissue. Boxplots of signal intensity distributions for each performed microarray are shown in Figure 2, indicating good data quality prior (blue) and after (red) normalization of the gene expression data (Figure 2A). A principal component analysis (PCA) was performed to show the common clustering of the triplicates (Figure 2B, blue, red and purple) as well as the differences of tested cell types, each represented by three different donors. We found that stromal cells from BM, adipose, and dental tissue are clearly distinct with respect to their transcriptomic profile. Interestingly, we detected a high donor-dependent variety of the gene expression for MSC derived from human BM (Figure 2B), suggesting a potential donor-specific impact on the efficacy of cardiac programming. A total of 1685 differentially expressed genes were detected, while 13 genes were shared by all MSC populations (Figure 2C). Most differentially expressed transcripts (679) have been found between MSCs obtained from BM and adipose tissue, suggesting a higher gene profile related diversity within these two MSC populations (Figure 2D). A list of differentially expressed genes between all MSC types is given in Table S1.

### 3.2. Reprogramming of MSC Using miRNA and Cardiac Induction Cell Culture Conditions

**Figure 3:**
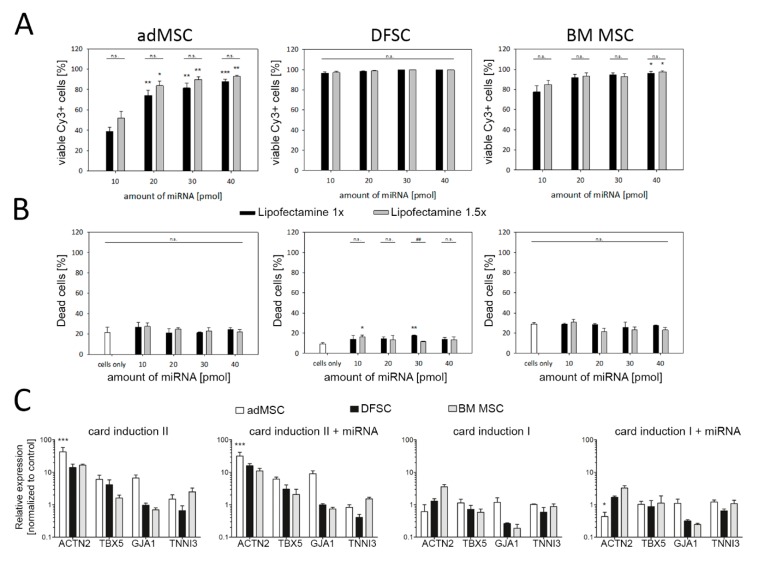
miRNA transfection and programming efficiency in MSC. (**A**) Uptake of miRNA was determined using Cy3-labelled miRNA and flow cytometry. (**B**) Detection of dead cells revealed low cytotoxicity induced by miRNA transfection. (**C**) Relative expression of cardiac marker genes among all tested cell types, four weeks after transfection and cultivation under different culture conditions. Reprogramming efficiency with cardiac induction medium I, II and myo-miRNAs (miR-1, miR-499, miR-208, miR-133) resulted in an up-regulation of cardiac specific markers in all types of MSC, while most profound up-regulation was found for cardiac induction medium II. Among tested MSC, the strongest increase of cardiac gene expression was observed for adMSC. Note, no beneficial effects on cardiac programming were observed following myo-miRNA transfection. Data are shown as mean ± SEM, obtained from three donors for each MSC type. Statistical analysis was performed using ANOVA test, followed by Bonferroni post-hoc analysis. * *p* ≤ 0.5, ** *p* ≤ 0.05, *** *p* ≤ 0.001.

In order to induce cardiac reprogramming, cells were cultured under two different medium conditions (see Section 2.3), separately or in combination with myocardial miRNAs (myo-miRNAs), that have been previously shown to induce cardiac differentiation in fibroblasts (miR-1, miR-499a, miR-208a, and miR-133a) [36]. As the efficiency of miRNA-based reprogramming largely depends on proper intracellular miRNA uptake, we evaluated miRNA transfection conditions using Cy3-labelled miRNA. Depending on the amount of transfected miRNA, uptake efficiencies of ~80–95% were achieved in all three cell types tested (Figure 3A). Importantly, only minimal cytotoxic effects were observed following transfection of miRNA (Figure 3B).

The success of myo-miRNA-based cardiac reprogramming was determined by qRT-PCR analysis of cardiac specific marker genes four weeks post transfection. Compared to control cells, cardiac induction medium II was found to be the most effective treatment leading to an induction of α-actinin, TBX5, GJA1, and cardiac Troponin I. While the level of α-actinin mRNA was strongly increased in all three cell types, a less pronounced effect was observed for cardiac Troponin I (Figure 3C). Notably, adMSCs showed the highest expression levels of cardiac marker genes after the treatment with cardiac induction medium II, when compared to MSCs obtained from dental follicle as well as BM, and therefore have been identified as the preferred candidate for our cardiac programming approach.

Surprisingly, our data also revealed that transfection with myo-miRNAs did not provoke an additional, beneficial effect on the expression of cardiac markers. Likewise, the cardiac induction medium containing RPMI and small molecules (Figure 3C, card induction I) did not promote the cardiac differentiation of MSC.

### 3.3. mRNA-Based Reprogramming of adMSC

As the transfection of miRNA did not further improve cardiac differentiation, we asked whether the application of modified mRNAs might boost the reprogramming efficiency in adMSC, which had been found to be the most promising cell type for the differentiation towards the cardiac lineage (Figure 3).

**Figure 4:**
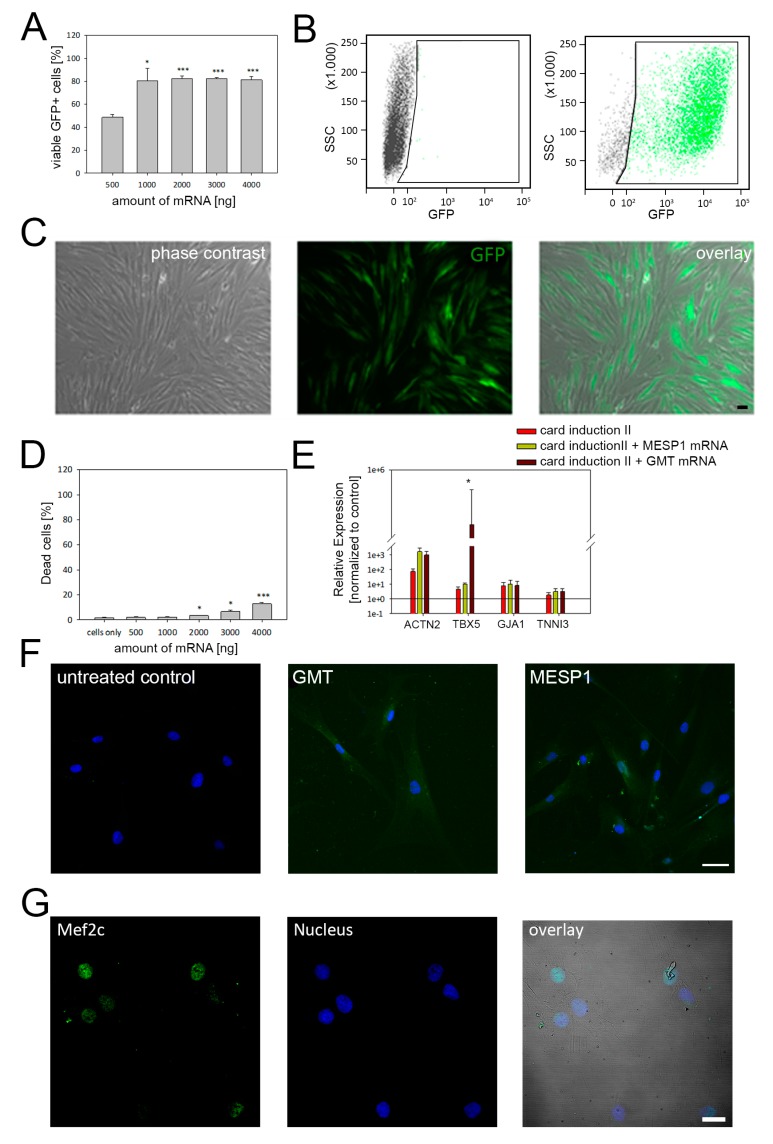
mRNA-based cardiac programming of adMSC. (**A**) Concentration-dependent expression of transfected mRNAs was evaluated with mRNA coding GFP. The quantative flow cytometry analysis demonstrated maximum transfection efficiency of ~80% when ≤ 1000 ng mRNA were applied. (**B**) Representative scatterplots of control cells (left) and cells transfected with GFP mRNA (right). (**C**) Corresponding microscopy images of cells expressing GFP following mRNA treatment. (**D**) Cytotoxic effects were only induced when mRNA amounts higher than 1000 ng were used for transfection. (**E**) Compared to untreated control cells, higher gene expression levels of selected cardiac markers were detected for all reprogramming conditions, in particular for α-actinin. (**F**) Immunolabeling of cells using anti α-actinin antibody results in a faint fluorescence signal in cells transfected with MESP1 and GATA4, MEF2C, and TBX5 (GMT) mRNAs, Scale Bar: 25 µm. (**G**) Moreover, GMT treated cells also demonstrated protein expression of MEF2C, an early cardiac transcription factor. Flow cytometry and qRT-PCR data are shown as mean ± SEM, obtained from three different donors. Statistical analysis was performed using one-way ANOVA. * *p* ≤ 0.5, ** *p* ≤ 0.05, *** *p* ≤ 0.001.

For mRNA-based programming of adMSC, cells were either transfected with single MESP1 mRNA or with a combination of GATA4, MEF2C, and TBX5 mRNA (GMT). First, mRNA transfection and translation efficiency were determined with mRNA encoding GFP to evaluate the optimal amount of mRNA showing strong expression while causing minimal cytotoxic effects. As demonstrated by flow cytometry and fluorescence microscopy, approximately 80% of cells express the GFP protein 24 h post transfection with 1 µg of mRNA (Figure 4A–C). Considering the increasing cytotoxicity when higher amounts of mRNA are transfected, reprogramming experiments were performed with 1–2 µg of individual mRNA (Figure 4D).

Analysis by qRT-PCR showed that both MESP1 and GMT transfection resulted in elevated levels of selected cardiac marker genes, compared to untreated control cells (Figure 4E). The most prominent incline of gene expression was observed for α-actinin, which was confirmed on the protein level by immunostaining showing a faint signal in cells treated with MESP1 and GMT mRNAs (Figure 4F). Additional antibody staining of early cardiac transcription factors demonstrated the expression of MEF2C and NKX2.5 on the protein level in GMT treated cells (Figure 4G and Figure S1). Interestingly, a profound increase of the expression level was also found for TBX5 that has been used for mRNA transfection in the GMT-treated group, verified by fluorescence microscopy (Figure S1).

Moreover, we observed differences of the intracellular Ca^2+^ concentration between treated groups. Following labelling of intracellular Ca^2+^, GMT transfected cells demonstrated a more intensive fluorescence signal than observed for MESP1 treated cells and the control group (Figure S2).

**Figure 5:**
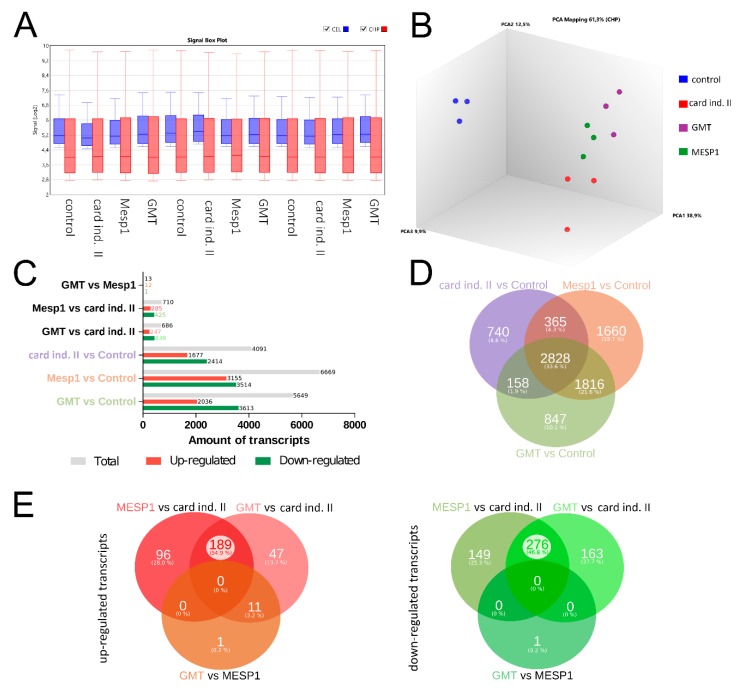
Transcriptome based comparison of reprogrammed adMSC. (**A**) Quality control of microarray data. Box plot of signal intensity of performed microarrays on .cel (blue) and .chp files normalization (red) confirm good data quality. (**B**) Principal component analysis (PCA) demonstrates clustering of treated groups, clearly showing the impact of respective reprogramming conditions on the transcriptomic profile compared to control cells (blue). Yet, cells subjected to MESP1 (green), GMT (purple) or cardiac induction medium II solely (red) remain distinguishable. (**C**) Up-and down-regulated transcripts and corresponding Venn diagram (**D**) showing the impact of reprogrammed cells compared to control. Most differentially expressed transcripts were regulated by all three reprogramming treatments (2828 genes), while 1816 transcripts are shared by GMT vs. control and MESP1 vs. control. (**E**) Detailed comparison of common and distinct up-regulated (red) and down-regulated (green) transcripts among the three reprogrammed groups. The differences found for optimized medium vs. MESP1 and GMT transfections are much more prominent than the differences between MESP1 and GMT.

To obtain a deeper understanding of the mRNA-induced effects on the gene expression profile of treated adMSC, we conducted a microarray analysis of cells that underwent cardiac reprogramming. The signal intensity values detected on each microarray had a similar spread after normalization, indicating a well-suited data quality for further downstream data analysis (Figure 5A). The PCA plot visualizes the differences in gene expression among treated groups, showing that control cells (blue) share a high similarity regarding their transcription profile (Figure 5B). In contrast, reprogramming with cardiac induction medium II (red), MESP1 (green), and GMT (purple) mRNA induced a strong donor-dependent alteration of gene levels, however, the treatment specific groups remain distinguishable from each other.

The numbers of significant total up-regulated and down-regulated transcripts are represented in Figure 5C, indicating a distinct change of gene expression following cardiac reprogramming. The highest number of genes differentially expressed was found in MESP1 (6669 transcripts) and GMT (5649) treated cells. Interestingly, more transcripts are down-regulated than up-regulated in most of the comparisons.

The corresponding Venn diagram (Figure 5D) compares the significantly expressed genes of the three different reprogramming approaches related to untreated control cells. The largest amount of transcripts (2828 transcripts, 33.6%) was found to be commonly regulated by all three treatments. The second largest proportion of differentially expressed genes is shared by GMT vs. Control and MESP1 vs. Control (1816 transcripts, 21.6%). Notably, the largest unique set of transcripts was found in cells transfected with MESP1 mRNA (1660 transcripts, 19.7%). A detailed comparison of up-regulated (Figure 5E, red) and down-regulated (Figure 5E, green) genes among these three reprogrammed groups indicates that the differences between MESP1 and GMT treatment vs. cardiac induction medium II are more profound (189 up-regulated, 276 down-regulated transcripts), while MESP1 and GMT only showed one differentially up-regulated transcript that was not previously up-regulated in other comparisons (Figure 5E). A detailed list of differentially expressed genes found in all reprogrammed groups is shown in in Table S1.

These data indicate a strong change of gene expression when cells are subjected to cardiac induction medium II, with more distinct effects induced by mRNA transfections.

**Figure 6:**
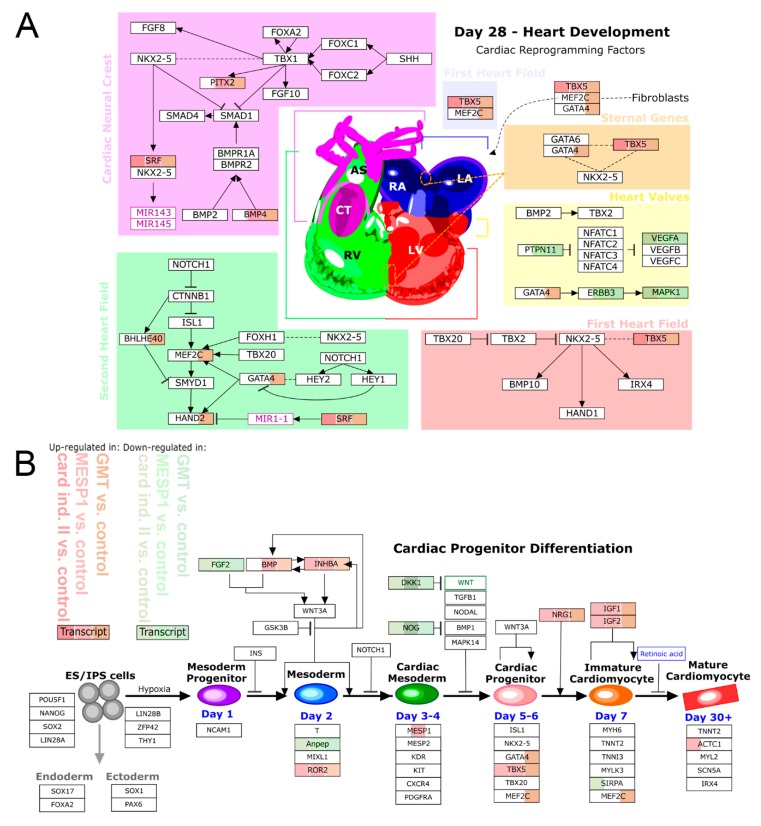
The impact of reprogramming on cardiac-differentiation pathways. Up-regulated and down-regulated transcripts of respective programming conditions are labelled in red or green color. (**A**,**B**) Strongest up-regulation of transcripts involved in cardiac development ((**A**) heart development, (**B**) cardiac progenitor differentiation) was mainly found in GMT reprogrammed cells, followed by MESP1 treatment and cardiac induction medium II. Key cardiac transcription factors and signaling molecules were significantly up-regulated, including TBX5, GATA4, MEF2C, HAND2, BMP4, and IGF.

To evaluate the influence of the differentially expressed genes on important cardiac development pathways, we integrated our microarray gene expression data into the WikiPathways database and identified significantly enriched pathways for “heart development” (Figure 6A) and “cardiac progenitor differentiation” (Figure 6B). The pathway visualization indicates proteins mainly involved in cardiac development, while up-regulated and down-regulated transcripts of respective programming treatments are labelled in red and green, respectively. As shown in Figure 6, cardiac induction medium II as well as mRNA programming by MESP1 and GMT influence the gene expression profile of several key transcription factors and signaling molecules involved in cardiac differentiation, such as IGF, VEGF, TBX5, GATA4 and HAND2 (Figure 6A,B). Most changes on pathway genes were induced by GMT treatment (92%), followed by MESP1 (60%) and cardiac induction medium (52%). Additional immunofluorescence labelling of GMT treated cells, confirmed the expression of early cardiac transcription factors, including NKX2.5, TBX5 and MEF2C (Figure 4G and Figure S1)

Taken together, the results obtained by microarray analysis clearly indicate that reprogramming with cardiac induction medium II and mRNA induced a strong alteration of the transcription patterns with high similarity in mRNA transfected cells compared to cells cultured in cardiac induction medium solely.

## 4. Discussion

In vitro generated cardiomyocytes are an important tool for cardiovascular research, as they can be utilized for disease modelling or for the development of drug screening assays to assess the cardiac toxic risk of established or newly synthesized drugs [37,38,39]. Moreover, promising preclinical data suggests the therapeutic potential of generated cardiomyocytes for the treatment of cardiac diseases to overall improve heart regeneration and function [40,41]. Although several stem cell types are available to produce cardiac cells, the ideal source of stem cells remains elusive as each has its own advantages and drawbacks. Adult MSC can be easily isolated from human donors in large quantities, possess immunomodulatory properties and can be propagated in vitro [12]. Further, they can overcome certain limitations that have been attributed to PSCs, including ESC and iPSC. In contrast to ESC, MSC do not provoke any ethical concerns [12,37,38]. Moreover, pre-clinical studies demonstrated a tumorigenic potential of ESC and iPSC-derived cell products that has not been observed for MSC to date [42,43,44,45]. However, other pre-clinical and clinical trial data showed that the transplantation of iPSCs-derived cardiomyocytes did not result in teratoma formation [46,47,48]. These different outcomes might be associated with the transplantation of residual undifferentiated cells along with the PSC product that increases the possibility of tumorigenesis. In this regard, the therapeutic use of PSC requires the establishment of differentiation protocols allowing the generation of highly pure PSC-derived cell types, e.g., cardiomyocytes [49]. The major advantage in comparison to adult stem cells is the cardiac differentiation potential of ESCs and iPSCs. So far, PSC have been found to be the only stem cell type capable to differentiate into functional, premature cardiomyocytes showing pronounced sarcomere organization, contraction capacity, and subtype specific ion channel composition [50,51]. Thus, for the generation of cardiomyocytes applied in regenerative medicine PSC are currently superior to MSC as no efficient cardiac reprogramming strategies have been developed for adult stem cells yet.

The successful cardiac differentiation of human MSC into fully mature cardiomyocytes is by far more challenging. Adult cardiomyocytes are characterized by a specific cell shape, structural organization, ion channel composition and mechanical properties; important features that need to be addressed when generating stem cell-derived cardiac cells [52]. Former reports led to contradictory results about the programming efficiency of MSC. While some reports described spontaneous beating associated with the formation of sarcomeric protein structures, other studies failed to generate cardiac-like cells from adult MSC [53,54,55,56,57].

One reason for this might be attributed to the fact that MSC may represent a heterogeneous stem cell population with different functional and phenotype-related properties as well as varying therapeutic potential [58]. A notion that is supported by our microarray data, indicating a high diversity of the expressed transcripts among MSC obtained from BM, dental pulp and adipose tissue (Figure 2). Likewise, our functional data revealed cell type-dependent differentiation capacity of tested MSC (Figure 1). Previous studies have also reported distinct characteristics between MSC from different sources regarding surface marker expression, proliferation rate, and differentiation potency [17,19,58,59]. For example, adMSC were observed to favor osteogenic differentiation and demonstrate higher proliferation when compared with DSFCs [18,60]. Moreover, our results suggest that these different biological characteristics of MSC could have an impact on the selected strategy and efficiency of cardiac programming as adMSC demonstrated a more pronounced incline of cardiac marker expression than BM MSC and DFSCs (Figure 3). In line with these data, Kakkar et al. recently described human adMSC to be a better choice for cardiac programming using a combination of small molecules and cytokines. Compared to BM MSC, adMSC exhibited a higher expression of α-actinin, troponin and connexin43 following cardiac induction with 5-Azacytidine and TGF-β1 [61]. Similarly, a comparative study revealed that adMSC expressed significantly more cardiomyocyte specific biomarkers as DFSCs following cardiac programming with cytokine supplemented culture medium [11]. The impact of MSC origin on programming capability was also shown for non-cardiac cell lineages like hepatocytes and smooth muscle cells [59,62].

Myo-miRNA based programming has been successfully applied for the conversion of cardiac fibroblasts, into cardiomyocytes [36]. For MSCs, cardiac induction by miRNA is less efficient as shown by different groups [25,63,64]. For example, it was demonstrated that transfection with miRNA-1-2 promote the expression of GATA4, NKX2.5 and cardiac Troponin in BM MSCs [15]. Similarly, miR-149 and miR-1 were found to slightly trigger myocardial differentiation, albeit without formation of sarcomere structures or beating activity [25,65]. We did not observe any additional effects on the expression of selected cardiac marker genes following miRNA treatment. This might be attributed to the fact that the miRNA concentrations used in this study are not sufficient to significantly increase the expression level of cardiac-specific genes, although uptake efficiency for miRNA was about 80%. In this regard, some studies have used viral vectors to ensure constitutive overexpression of miRNA [25,64]. Given that miRNAs have a very short half live, transient transfection approaches, as used in our study, might be less effective.

Proper cardiac development requires the activation and inhibition of many different pathways modulated by several transcription factors [66]. MESP1 was shown to drive cardiovascular fate of stem cells during embryonic development, while the combination of GATA4, MEF2C and TBX5 was described to induce the cardiac differentiation of murine and human fibroblasts, leading to spontaneously contracting cells with cardiomyocyte-like expression profile [67,68,69,70]. Therefore, we have concluded that this approach might be applicable to reprogram human adMSC. Using an mRNA-based setting we induced the overexpression of GATA4, MEF2C, and TBX5 as well as MESP1, which provoked an incline of genes involved in cardiac differentiation (Figure 4). To our knowledge this combination of transcription factors has not been applied before to induce cardiac differentiation of human adMSC. In contrast to our strategy, most of the previous studies performed overexpression of transcription factors by application of retro- or lentiviral systems. For example, in a study by Wystrychowski et al., adMSC from cardiac tissue were treated with seven transcription factors, including GATA4, MEF2C, MESP1, and TBX5, that resulted in an elevated number of cells positive for α-actinin and troponin [71]. However, no clear sarcomere structures have been observed, suggesting a premature cardiac progenitor state. Similarly, forced expression of another factor of the T-box family, TBX20, provokes an up-regulation of sarcomeric proteins, without cardiomyocyte specific sarcomere organization [72]. These data are in line with our observations as we could also detect a moderate signal for α-actinin, albeit without the presence of sarcomere structures (Figure 4).

Yet, our programming approach leads to a strong induction of the key cardiac transcription factors GATA4, MEF2C, MESP1 and TBX5, which corresponds to the transfected mRNAs used for programming. However, it is known that mRNAs underlie fast turnover, suggesting that mRNA transfection activated the expression of its endogenous counterparts [73,74]. At the same time, the current study demonstrates that mRNA transfection boosts the cardiac programming effects induced by culture conditions targeting important signaling pathways such as the WNT cascade.

The manipulation of signaling pathways by cytokines and small molecules is the most common methodology to generate large amounts of PSC-derived functional cardiomyocytes [30,31]. In addition, the overexpression of transcription factors, like Tbx3 and MESP1, can influence cell fate decision in PSCs [75,76]. While these techniques allow highly efficient programming of ESCs and iPSCs, we observed significantly less programming efficiency for MSCs in the current study. However, the comparison of programming protocols used for PSCs and multipotent stem cells is difficult due to their different developmental stages and resulting culture conditions prerequisites. Yet, it was shown that cytokines like BMP4, IL and TGF improve cardiac development of human and non-human MSCs [57,77]. However, the cardiomyocyte-like cells derived from these programmed MSCs lack profound sarcomere formation, beating activity and physiological maturation [78,79]. This is in accordance to our data indicating that mRNA transfection could promote the expression of early cardiac proteins, while differentiation efficiency and elaboration of a terminal cardiac phenotype is profoundly limited when compared to PSC differentiation protocols [27,31].

Together with previous studies of adMSC overexpressing transcription factors, our results demonstrate the feasibility of mRNA-based cardiac reprogramming of MSC. However, the absence of sarcomere structures and spontaneous cell beating suggests a yet quite incomplete reprogramming, leading to an immature cardiac cell type. Hence, there is an urgent need for further optimization. Since mRNAs are degraded over time, multiple transfection steps might increase the reprogramming efficiency, a strategy that is already applied for the generation of iPSCs from adult cells [74,80]. Moreover, proportions of GATA4, MEF2C, and TBX5 protein expression has been described to play a crucial role for the quality of cardiac reprogramming [81], thus, different ratios of transfected mRNA could positively influence the outcome of reprogrammed adMSC. This will have to be addressed in future studies as the impact of mRNA ratios and mRNA concentration on cardiac programming might be affected in a donor specific manner. Former data already demonstrated donor-to-donor variability of MSC functional potential, including differentiation capacity [82,83]. Beside age and gender, underlying diseases are known to influence cellular properties of MSCs [82]. This is supported by our microarray results, showing a large variety of the transcription profile of BM MSCs that have been obtained from patients suffering from cardiovascular diseases. On the contrary, adMSCs and DFSCs derived from healthy donors shared similar transcription patterns, suggesting same programming conditions required to induce cardiac development. Nevertheless, it is recommended to adapt mRNA conditions for each individual patient to obtain maximum programming efficiency.

In addition, more comparative studies are required to identify and characterize MSC subtypes most susceptible for specific transdifferentiation towards the respective desired target cells, including non-mesodermal and mesodermal cell types such as cardiomyocytes.

## Supplementary Materials

The following are available online at https://www.mdpi.com/2073-4409/9/2/504/s1.
